# Burrow Dusting or Oral Vaccination Prevents Plague-Associated Prairie Dog Colony Collapse

**DOI:** 10.1007/s10393-017-1236-y

**Published:** 2017-06-22

**Authors:** Daniel W. Tripp, Tonie E. Rocke, Jonathan P. Runge, Rachel C. Abbott, Michael W. Miller

**Affiliations:** 1 Colorado Division of Parks and Wildlife, Wildlife Health Program, 4330 Laporte Avenue, Fort Collins, CO 80521-2153 USA; 2 United States Geological Survey, National Wildlife Health Center, 6006 Schroeder Road, Madison, WI 53711 USA; 3 Colorado Division of Parks and Wildlife, Terrestrial Resources Program, 317 West Prospect Road, Fort Collins, CO 80526-2097 USA

**Keywords:** Black-tailed prairie dog, *Cynomys ludovicianus*, Deltamethrin, Plague, Raccoonpox, Vaccine

## Abstract

Plague impacts prairie dogs (*Cynomys* spp.), the endangered black-footed ferret (*Mustela nigripes*) and other sensitive wildlife species. We compared efficacy of prophylactic treatments (burrow dusting with deltamethrin or oral vaccination with recombinant “sylvatic plague vaccine” [RCN-F1/V307]) to placebo treatment in black-tailed prairie dog (*C. ludovicianus*) colonies. Between 2013 and 2015, we measured prairie dog apparent survival, burrow activity and flea abundance on triplicate plots (“blocks”) receiving dust, vaccine or placebo treatment. Epizootic plague affected all three blocks but emerged asynchronously. Dust plots had fewer fleas per burrow (*P* < 0.0001), and prairie dogs captured on dust plots had fewer fleas (*P* < 0.0001) than those on vaccine or placebo plots. Burrow activity and prairie dog density declined sharply in placebo plots when epizootic plague emerged. Patterns in corresponding dust and vaccine plots were less consistent and appeared strongly influenced by timing of treatment applications relative to plague emergence. Deltamethrin or oral vaccination enhanced apparent survival within two blocks. Applying insecticide or vaccine prior to epizootic emergence blunted effects of plague on prairie dog survival and abundance, thereby preventing colony collapse. Successful plague mitigation will likely entail strategic combined uses of burrow dusting and oral vaccination within large colonies or colony complexes.

**Electronic supplementary material:**

The online version of this article (doi:10.1007/s10393-017-1236-y) contains supplementary material, which is available to authorized users.

## Introduction

Plague—caused by the bacterium *Yersinia pestis*—impacts numerous wildlife species worldwide. Its introduction has contributed to the degradation of North American grassland and shrub-steppe ecosystems (Gage and Kosoy 2005; Augustine et al. 2008; Eads and Biggins 2015). Prairie dogs (*Cynomys* spp.) in particular suffer plague-driven mass mortality that can collapse colony complexes over large geographic areas (e.g., Ecke and Johnson 1952). Other associated wildlife species, like the endangered black-footed ferret (*Mustela nigripes*) that rely on prairie dogs for habitat or prey, may be directly or indirectly affected by plague (Antolin et al. 2002; Biggins et al. 2010). The ability to mitigate plague at an ecologically meaningful scale has thus emerged as a critical conservation need (Creekmore et al. 2002; Seglund and Schnurr 2010; Biggins et al. 2010; Abbott et al. 2012).

Until recently, the plague management approach most widely practiced in North America was reactive use of insecticides to control fleas, the primary plague vector (Seery et al. 2003; Biggins et al. 2010). This approach can be effective in reducing mortality and spillover to domestic animals and humans but does little to offset the broader ecological impacts of epizootic plague. Since the early 2000s, attention has shifted to developing preventive plague management approaches for prairie dog habitats via vector control (Hoogland et al. 2004; Biggins et al. 2010; Griebel 2012; Jachowski et al. 2012; Tripp et al. 2016) and oral vaccination (Mencher et al. 2004; Rocke et al. 2010, 2014; Abbott et al. 2012).

Here, we describe a field experiment designed to assess and compare the effectiveness of annual burrow dusting or oral vaccination in preventing plague in a black-tailed prairie dog (*C. ludovicianus*) colony complex. Our study provides insights into the benefits and limitations in field application of two specific plague management tools: deltamethrin dust (Seery et al. 2003; Biggins et al. 2010; Tripp et al. 2016) and a raccoonpox-vectored plague vaccine designated “sylvatic plague vaccine” or SPV (Abbott et al. 2012; Rocke et al. 2014; Tripp et al. 2015). Our observations also more broadly inform on developing adaptive management strategies intended to prevent widespread, plague-induced mortality among prairie dogs.

## Methods

This study was conducted during Aug 2012–Oct 2015 as a collaboration of the Colorado Division of Parks and Wildlife (CPW), the U.S. Geological Survey (USGS) National Wildlife Health Center (NWHC) and the City of Fort Collins. The CPW Animal Care and Use Committee (file number 05-2012 and 06-2013) approved study protocols. Field use of vaccine was approved by U.S. Department of Agriculture’s Center for Veterinary Biologics (USDA CVB), and an environmental assessment of vaccine use was completed by the USGS (2012) with a finding of no significant impact by USDA CVB (http://www.nwhc.usgs.gov/disease_information/sylvatic_plague).

### Study Area and Design

**Figure 1:**
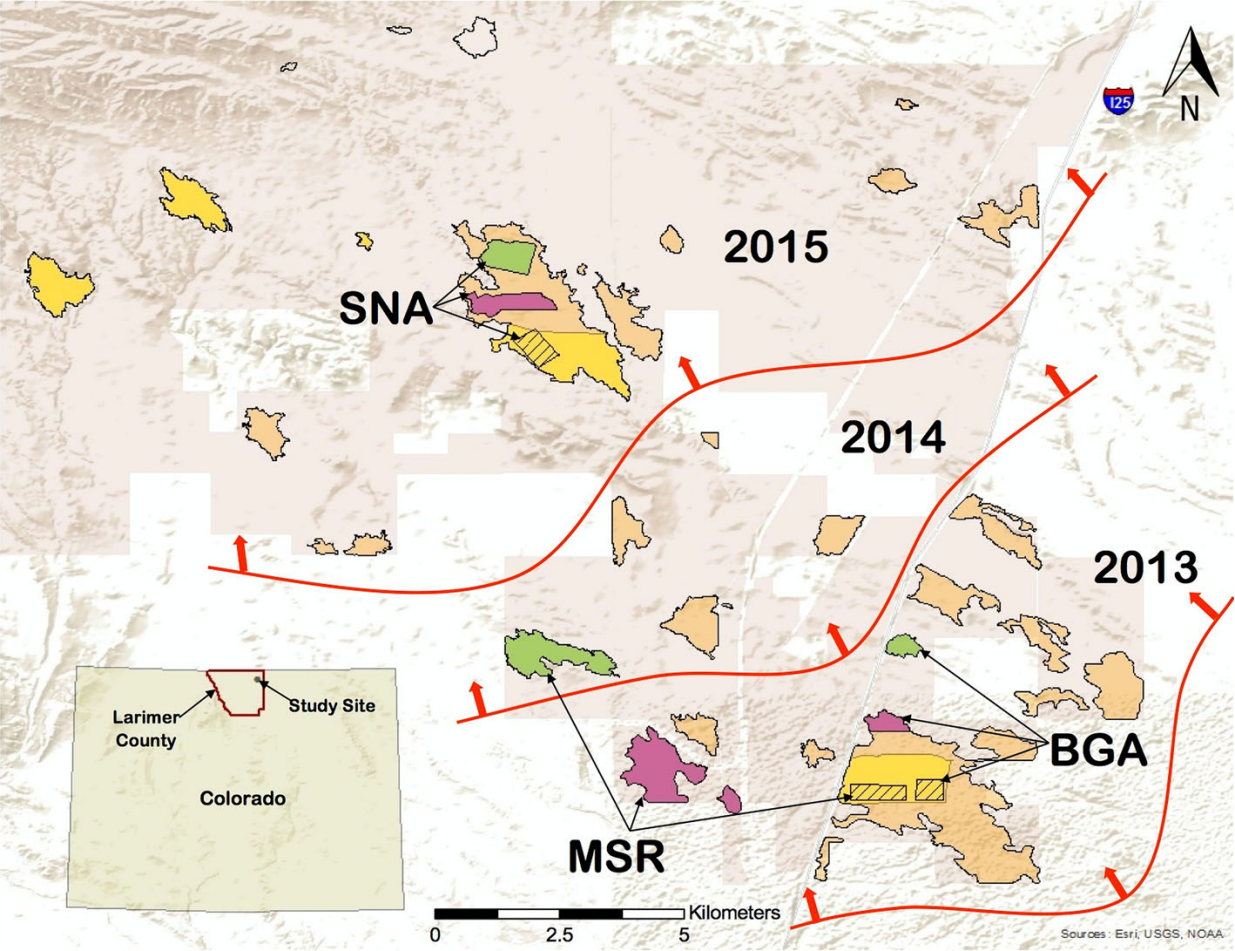
Map of the study area: Bulger Grazing Allotment (BGA), Meadow Springs Ranch (MSR), Soapstone Natural Area (SNA) study blocks in Larimer County Colorado. Black-tailed prairie dog (*Cynomys ludovicianus*) colonies are shown in their largest extent during 2012–2015. Prairie dog colonies impacted by plague during the study are tan. Areas treated with insecticidal dust (*yellow*, study plots are crosshatched), vaccine baits (*purple*) and placebo baits (*green*) are shown. The northeasterly spread of epizootic plague during the study is represented by *red lines* and *arrows* with the year of colony collapse shown.

We established nine treatment plots within a 1375 ha black-tailed prairie dog colony complex (Soapstone–Meadow Springs) located on natural areas in northern Larimer County, Colorado, USA, owned and managed by the City of Fort Collins (Figure 1). Epizootic plague had been last documented in this complex during 2008–2009 (Griffin et al. 2010). We compared three treatments (insecticidal dust, vaccine, placebo) in triplicate. Within each dust–vaccine–placebo block (Bulger Grazing Allotment [BGA], Meadow Springs Ranch [MSR], Soapstone Natural Area [SNA]), we clustered plots closely (Figure 1) to minimize potential confounding effects of spatiotemporal variation in plague activity across the complex. We assigned an entire colony to a single treatment in four cases. In five cases, where we established plots within a larger colony, we buffered boundaries by ≥400 m to minimize spillover between treatments (Table 1; Figure 1). Plot location and size, as well as treatment assignments, were somewhat constrained by other land use priorities, vaccine availability and prior plague mitigation activities (Table 1). Of particular note, vaccine and placebo plots at MSR and SNA were dusted in 2012 as part of a vaccine safety trial or during prior plague management programs (Tripp et al. 2015; Table 1). The BGA and MSR dust plots were spatially adjacent and located within a larger dusted block (Figure 1) but were regarded as independent. We do not believe that these constraints unduly biased overall outcomes.

**Table 1:** Summary Information About Study Blocks and Plots, Treatment Application, Prior Plague Management History, Bait Uptake and Plague Detection During 2013–2015 Study Period.

Block Treatment plot	Month treated	Plot/colony size (ha)	Prior dusting history	Bait uptake^a^ (%) (2013, ’14, ’15)	Plague activity first detected (Mo–Yr)		
					Burrow flea	On-host flea	Carcass
Bulger Grazing Allotment (BGA)							
Dust	Mar/Apr	Plot/16	Mar/April 2010–15	nb	None	None	None
Vaccine	Aug	Colony/16	None	55, 0, 33	Aug 2013	Aug 2013	Aug 2013
Placebo	Aug	Colony/16	None	63, 33, 73	Mar 2014	Oct 2013	None
Meadow Springs Ranch (MSR)							
Dust	Mar/Apr	Plot/24	Mar/April 2010–15	nb	None	None	None
Vaccine	Aug	Colony/53^c^	Sep 2011, May 2012	94, 93, 91	Apr 2014	Sep 2013	Aug 2013
Placebo	Aug	Colony/74^c^	Sep 2011, May 2012	75, nc, 33	Jul 2014	Sep 2014	Jun 2014
Soapstone Natural Area (SNA)							
Dust	Mar/Apr	Plot/40	Mar/April 2010–15	nb	Mar 2015	None	None
Vaccine	Aug	Plot/40	Mar/April 2010–12	96, 88, 98	Mar 2015	Jun 2015	Jun 2015
Placebo	Aug	Plot/40	Mar/April 2010–12	85, 84, nc	Jul 2014	Jun 2015	Jun 2015

^a^ *nb* not baited, *nc* none captured.

^b^Black-footed ferrets released on or near plot (Mo–Yr).

^c^8 ha were baited in 2012. See Tripp et al. (2015) for details.

### Plot Treatments

Insecticidal “dust” plots were established within select prairie dog colonies treated annually during March–April by City of Fort Collins Natural Areas Program staff since 2010. Crews used specialized applicators (Technicide, San Clemente, CA) to deliver 4–5 g of 0.05% deltamethrin powder (DeltaDust^®^, Bayer Crop Science, Research Triangle Park, North Carolina; Seery et al. 2003; Biggins et al. 2010; Tripp et al. 2016) into each prairie dog burrow encountered.

Vaccine and placebo treatments described here were also part of a broader multistate effort to evaluate the field efficacy of SPV (Rocke et al. 2017). “Vaccine” plots received baits carrying recombinant raccoonpox virus (RCN-F1/V307; unlicensed Yersinia pestis Vaccine, Live Raccoon Poxvirus Vector, Code 11Y2.R0; Rocke et al. 2014). Each ~4–5 g bait carried about 5 × 10^7^ plaque-forming units of RCN-F1/V307 and consisted of an edible polymer (Food Source Lures, Alabaster, Alabama, USA) and peanut butter, with rhodamine B (0.25%) incorporated as a biomarker (Fernandez and Rocke 2011; Tripp et al. 2014, 2015). We distributed baits by hand along transects spaced 10 m apart, dropping one every ~10 m for an application rate of 99 baits/ha in 2012–2013 or 124 baits/ha in 2014–2015 following methods described by Tripp et al. (2014, 2015). Baits were distributed on vaccine plots each August after juveniles had emerged from natal burrows (and when cattle were absent) to maximize uptake by prairie dogs (Tripp et al. 2014). Vaccine distribution began in 2013, except for an 8 ha subplot in the MSR block that had been treated in 2012 (Table 1; Tripp et al. 2015). “Placebo” plots received baits identical in composition but without RCN-F1/V307; these were applied as described for vaccine. All vaccine and placebo baits were made at NWHC following methods described by Rocke et al. (2017).

### Indices of Prairie Dog Abundance and Survival

We conducted burrow counts, capture, sampling and marking of prairie dogs on all plots within each block using the same schedule and procedures to maintain consistency for comparisons.

Prairie dog capture began 6–20 days after bait distribution to measure and compare bait uptake. The capture schedule followed a robust design method (Pollock et al. 1990; Kendall et al. 1995) to estimate abundance and survival. The robust design specifies that multiple consecutive (or near consecutive) trapping occasions (i.e., trap days) compose a multi-day primary trapping session and that primary sessions are repeated. An interval of approximately 1 year separated trapping sessions in this study, except that two sessions were conducted opportunistically at BGA in 2013 and SNA in 2015 as plague emerged. Survival was estimated for the intervals between trapping sessions (primary period), and abundance and density were estimated from a minimum of four trapping/marking occasions (days) within each session (Otis et al. 1978; Pollock et al. 1990; Gould and Pollock 1997). We calculated density by dividing abundance estimates by effective area trapped, which was the area trapped with a 20 m buffer (half of the estimated home range of a prairie dog) to account for individuals captured on the perimeter. We marked all prairie dogs that were captured during trapping.

### Animal Capture and Handling

Prairie dog capture, handling and marking generally followed methods described elsewhere (Tripp et al. 2009, 2014, 2015, 2016; detailed in Supplemental Material). We captured prairie dogs on at least four consecutive days but anesthetized and sampled individuals only once per trapping session.

### Plague Surveillance

We collected fleas from 90 randomly selected burrows at all study plots in May, July and September throughout the study. Prairie dog burrows were classified as active or inactive (Biggins et al. 1993) and swabbed using methods modified from Ecke and Johnson (1952) as described elsewhere (Griffin et al. 2010; Tripp et al. 2015, 2016; detailed in Supplemental Material). We also collected fleas from captured prairie dogs (detailed in Supplemental Material).

### Laboratory Analyses

All fleas were identified to the species level using the keys of Stark (1958) and Hubbard (1968). Fleas of a single species from individual prairie dogs and burrows were placed into pools of up to 10 fleas and tested for *Y. pestis* by PCR (Griffin et al. 2010). Prairie dog and other carcasses were retained for necropsy and plague screening via PCR (Griffin et al. 2010) using the Qiagen supplementary protocol for liver and spleen tissue and the DNeasy blood and tissue kit (Qiagen, Valencia, California, USA); the US Centers for Disease Control and Prevention (Fort Collins, Colorado, USA) confirmed positive carcass results with direct fluorescent antibody (DFA) and mouse inoculation tests. To assess bait uptake, hairs and whiskers from live-trapped prairie dogs were examined under a fluorescence microscope (Fernandez and Rocke 2011).

### Data Analyses

We compared flea abundance between plots using Wilcoxon rank-sum tests with *P*-values adjusted by the Holm (1979) procedure. The R statistical base package (pairwise.wilcox.test) was used for these analyses (R Development Core Team, Version 2.15.2 2012). We compared burrow activity between plots using Chi-square tests (prop.test).

We used program MARK (Burnham and Anderson 1998) to estimate abundance and probabilities of apparent survival, capture and recapture. We used the Huggins robust design model (Pollock et al. 1990; Huggins 1991) to estimate parameters of interest in the BGA and MSR blocks. The SNA block had sufficient data to support a multistate robust design model (Nichols and Coffman 1999). We fit models with the following variables: sex, estimated age (animals ≤15 months old vs. >15 months old in multistate robust design models; animals first marked at ≤15 mo old vs. first marked >15 mo old in robust design only models), primary period, treatment (dust, vaccine or placebo), proportion of colony trapped, and whether the plot had experienced epizootic plague as defined by the observed decline of the colony (>90% reduction in prairie dog numbers).

Fitting all possible models and combinations for both survival and recapture would have resulted in a set of over 1000 models; therefore, model selection was conducted according to methods detailed in Supplemental Material. ΔAICc (Akaike Information Criteria; Burnham and Anderson 2002) is the difference between the lowest-AICc model and the model referenced; AICc weight is the relative weight or belief assigned to each model. We show models with ΔAICc ≤ 10.

## Results

### Plague

**Figure 2:**
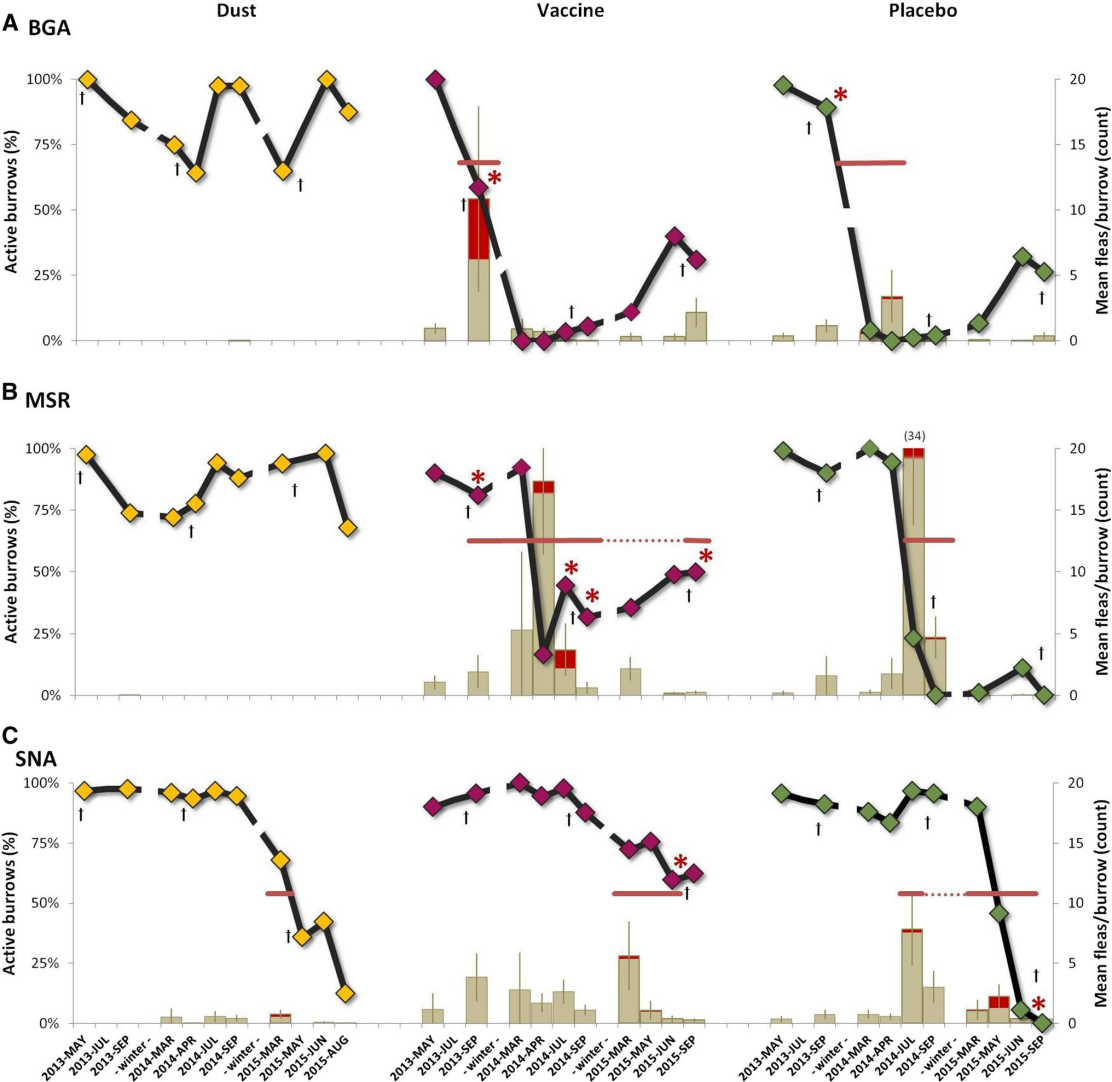
Black-tailed prairie dog (*Cynomys ludovicianus*) burrow activity (active burrows/total burrows scored) on the individual study areas (A. BGA, B. MSR, C. SNA; *black lines*, left axis). Fleas were collected from prairie dog burrows in May, July and September 2013–2015 in areas that received deltamethrin dust, vaccine or placebo baits. Breaks in the *lines* represent overwinter periods when sampling was not conducted. Mean flea abundance in prairie dog burrows is shown (*bars*, right axis); the portion of each bar shaded in *red* represents the overall proportion of flea pools (from prairie dog burrows) positive for *Yersinia pestis* DNA at each plot-sampling point, shown as an index of relative plague activity. The duration of sustained plague activity (*red horizontal bars*) and sporadic plague activity (*red dotted red lines*) is shown. Sampling periods in which *Yersinia pestis*-positive carcasses or on-host fleas were collected are shown as red asterisks. *Dagger* represents the month of treatment application. All *error bars* are 95% confidence intervals.

**Figure 3:**
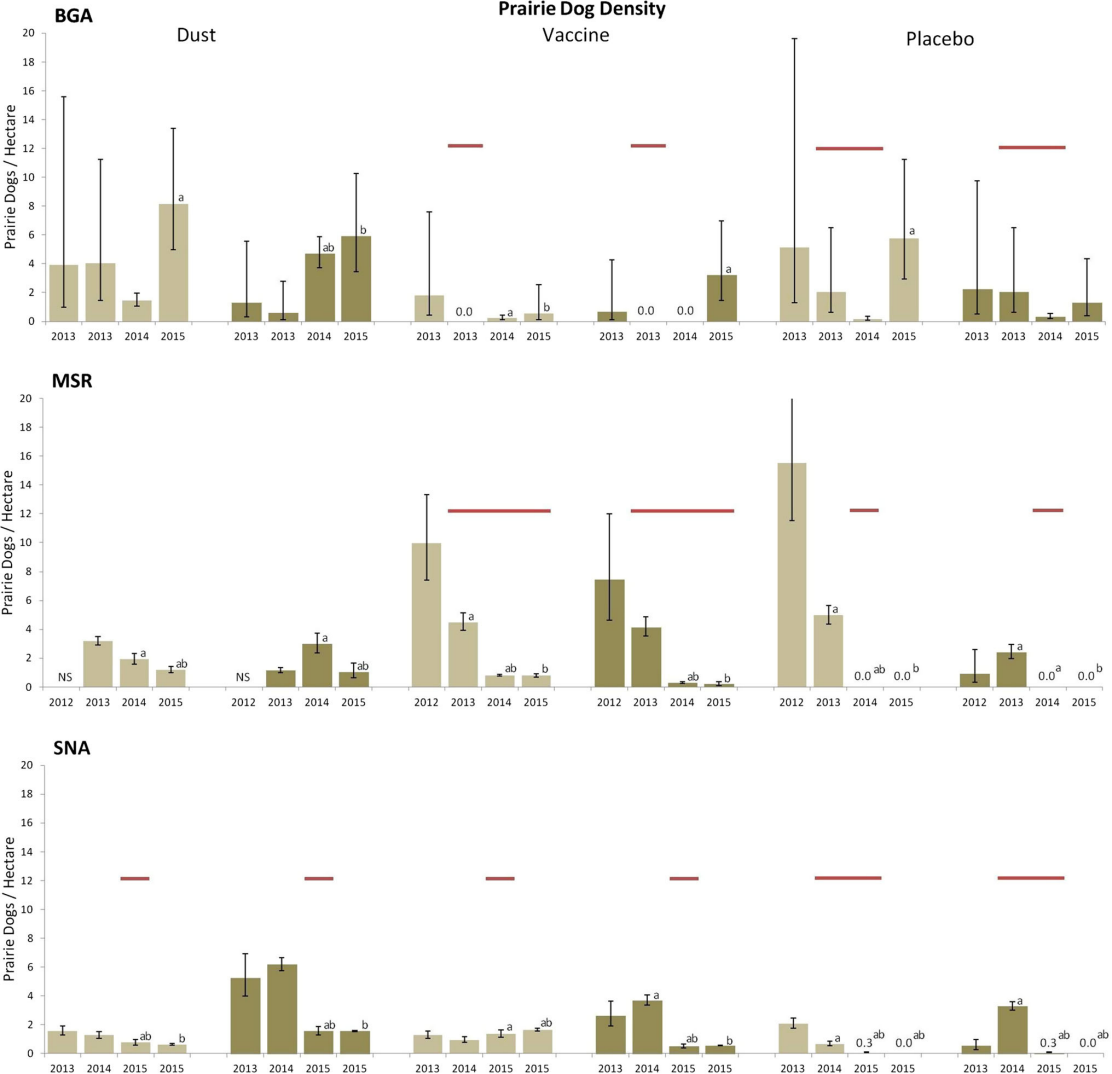
Density of adult (*light bars*) and young (*dark bars*) black-tailed prairie dogs (*Cynomys ludovicianus*) per hectare on dust, vaccine and placebo plots on the Bulger Grazing Allotment (BGA), Meadow Springs Ranch (MSR) and Soapstone Natural Area (SNA) study blocks. Density is defined as mark–recapture abundance estimates/effective area trapped in hectares. The duration of plague activity (*red horizontal bars*) is shown. All *error bars* are 95% confidence intervals. Differences (95% confidence intervals do not include zero) in prairie dog density between the current and prior primary capture session (**a**), and between the current and the two prior primary capture sessions (**b**) are noted.

Epizootic plague affected all three blocks during the study period but emerged asynchronously among and within blocks (Table 1; Figure 1). We detected plague in fleas and prairie dog carcasses in the southeastern portion of the complex as our field experiment began in August 2013 (Table 1; Figure 1). The epizootic subsequently spread in a northwesterly direction, reaching the northern portion of the complex in 2015 (Table 1; Figure 1). Burrow activity and prairie dog density declined sharply in placebo plots at all three blocks when epizootic plague emerged (Figures 2, 3; Table S1).

Patterns in the corresponding dust and vaccine plots were less consistent and appeared strongly influenced by the timing of treatment applications relative to plague emergence (Table 1; Figure 2). Temporal asynchrony in epizootic plague emergence and its strong effects on prairie dog dynamics rendered most planned pooled comparisons uninformative. Consequently, we report analyses and patterns for each block separately in chronological order of plague emergence (BGA, MSR, SNA).

### Burrow Activity

We first detected plague at the BGA block in August and October 2013 on the vaccine and placebo plots, respectively (Table 1; Figure 2). Plague detection portended rapid declines in burrow activity on both of these plots (Figure 2). Placebo plot burrow activity collapsed (= 0%) by May 2014 and remained low through October 2015 (range 1–32%; Figure 2). Burrow activity on the vaccine plot also collapsed by March 2014 and remained low (0–40%; Figure 2). In contrast, we recovered no plague-positive fleas or carcasses on the BGA dust plot and prairie dog burrow activity remained high (64–100%; Figure 2). In March 2014, burrows in the dust plot were ≥71% more active (Chi-square test; *P* < 0.0001) than in the vaccine or placebo plots (which did not differ; *P* = 0.4751).

The pattern at the MSR block was similar. We first detected plague in August 2013 on the vaccine plot and in June 2014 on the placebo (Table 1). Rapid declines in burrow activity followed (Figure 2). Burrow activity on the placebo plot collapsed by September 2014 and remained low (0–11%; Figure 2). Burrow activity on the vaccine plot decreased to 16% by April 2014 but rebounded to between 32 and 62% thereafter (Figure 2). Like BGA, we recovered no plague-positive fleas or carcasses on the MSR dust plot, and burrow activity remained high (68–98%; Figure 2). In September 2014, burrow activity in the MSR dust plot was 56% greater (*P* < 0.0001) than in the vaccine or placebo plots; activity in the vaccine plot (32%) was greater than in the placebo plot (0%; *P* < 0.0001).

Burrow activity remained relatively high at the SNA block until 2015. We first detected plague on the placebo plot in July 2014 and on the dust and vaccine plots in March 2015 (Table 1). Declines in burrow activity preceded or followed plague detection (Figure 2). Placebo plot burrow activity decreased to 6% by June 2015 and collapsed by September. Dust plot burrow activity decreased to 12% by September 2015. Burrow activity on the vaccine plot declined less dramatically, with 60% remaining active in June 2015. In September 2015, 50% fewer burrows were active in the dust plot (*P* < 0.0001) than in the vaccine plot but 12% more burrows were active (*P* = 0.0019) than in the placebo plot; 62% more burrows were active on the vaccine plot than on the placebo plot (*P* < 0.0001).

### Survival and Density

Plague’s emergence in the BGA block preceded or coincided with the beginning of our study, confounding survival analyses. The lowest-AICc model (weight ~0.9; Table 2) indicated differences in apparent survival (*S,* accounts for survival and emigration), capture (*c*) and recapture (*p*) probabilities among primary periods with c and p being unequal. In the nearest ranked model (ΔAICc = 5.5; weight ~0.06; Table 2), *S* changed differently among age groups across primary periods. Estimated monthly *S* was 0.55 (SE = 0.24) between August and October 2013, then ≥0.82 (0.04) for the remainder of the study (Table S2). Except for the 2-day October 2013 primary period in which no recaptures occurred, *c* ≥ *p* (Table S2). Estimated densities on the BGA dust plot were generally higher and more stable than either vaccine or placebo plots, both of which collapsed by 2014 (Figure 3, Table S1). Prairie dog densities were higher throughout the experiment on the placebo colony than on the vaccine colony (Figure 3). This is likely because the former remained occupied to some extent, whereas the latter was temporarily devoid of prairie dogs after the epizootic. Thus, recovery occurred more rapidly on the placebo colony.

**Table 2:** Model Selection Results from the Bulger Grazing Allotment, Meadow Springs Ranch and Soapstone Natural Area Study Blocks.

Models selected by block	AICc	ΔAICc	AICc weight	*k*
Bulger Grazing Allotment				
*S*(period) *p*(period) *c*(period) *p* ≠ *c*	1307.76	0	0.896	11
*S*(age * period) *p*(period) *c*(period) *p* ≠ *c*	1313.27	5.51	0.057	14
*S*(sex * period) *p*(period) *c*(period) *p* ≠ *c*	1313.82	6.06	0.043	14
Meadow Springs Ranch				
*S*(treatment * period) *p* = *c*(age * period)	4783.06	0	0.567	14
*S*(age * period + treatment) *p* = *c*(age + period)	4784.14	1.08	0.330	13
*S*(age * period + treatment) *p* = *c*(period)	4786.71	3.65	0.091	12
*S*(age + period + treatment) *p* = *c*(age + period)	4792.33	9.27	0.006	11
*S*(age * period + treatment) *p* = *c*(age * period)	4792.63	9.57	0.005	16
Soapstone Natural Area				
*S*(period * treatment) *p* = *c*(age * period)	4249.46	0	0.817	17
*S*(treatment) *p* = *c*(age * period)	4252.81	3.35	0.153	11
*S*(age * period * treatment) *p* = *c*(age * period)	4256.30	6.85	0.027	26

Only models within 10 AICc (Akaike Information Criteria) units of the low-AICc model are shown.

ΔAICc is the difference between the lowest-AICc model and the model referenced; AICc weight is the relative weight or belief assigned to each model; *k* is the number of parameters in the model; *S* is apparent survival, *p* is initial capture probability, *c* is recapture probability, *p* = *c* indicates that the two are equal, *p* ≠ *c* indicates the two are estimated separately, treatment represents differences across the three treatment types (dust, vaccine or placebo), period represents differences among estimates between primary periods (trapping sessions).

Plague management showed stronger effects in the MSR and SNA blocks. The low-AICc model for each suggested that *S* varied by treatment and primary period and that *p* varied by age and primary period, with *p* = *c* (weights ~0.57 and ~0.82, respectively; Table 2).

**Figure 4:**
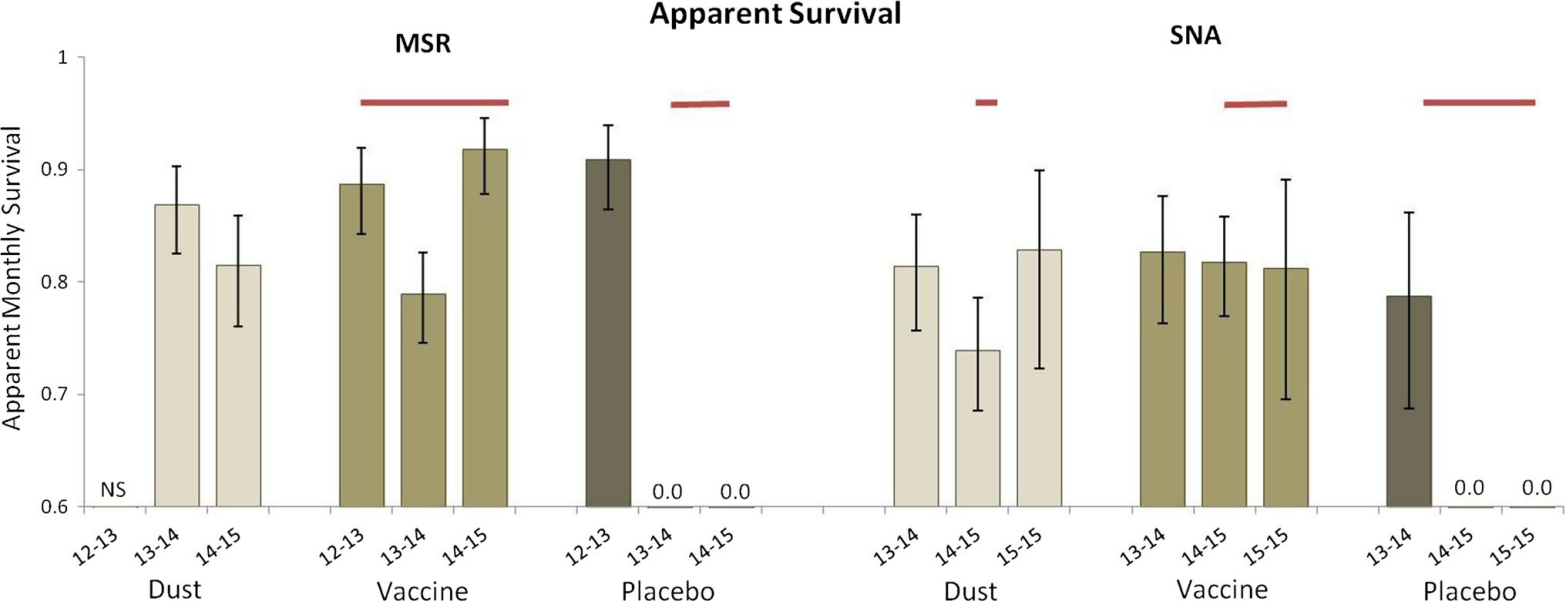
Apparent monthly survival of black-tailed prairie dogs (*Cynomys ludovicianus*) on dusted, vaccine and placebo plots on the Meadow Springs Ranch (MSR) and Soapstone Natural Area (SNA) study blocks is shown. Survival data from the Bulger Grazing Allotment (BGA) study block were insufficient for comparison between treatments. The duration of plague activity (*red horizontal bars*) is shown. All *error bars* are 95% confidence intervals. NS = not sampled in 2012. See Tripp et al. (2015) for details of sampling/marking at MSR in 2012.

At MSR, all five lowest-scoring AICc models (ΔAICc ≤ 10; combined weight ~0.998; Table 2) included an effect of dust or vaccine on survival. The MSR block had sufficient data to fit multistate robust design models. We did not trap on the dust plot prior to 2013, but monthly *S* was estimated at ≥0.81 (0.03) throughout the study (Table S2; Figure 4). Monthly *S* in the vaccine plot was estimated at 0.89 (0.02) between September 2012 and September 2013 for animals marked by Tripp et al. (2015), then declined to 0.79 (0.02) before rebounding (0.92 ± 0.02; Figure 4; Table S2). Estimated monthly *S* in the placebo plot was 0.91 (0.02) between September 2012 and September 2013 for animals marked by Tripp et al. (2015), but 0.0 (0.0) after epizootic plague emerged (Figure 4; Table S2). Capture rates for adults were slightly higher than for young (Table S2); capture and recapture rates were equal.

The three lowest-scoring AICc models for SNA (combined weight ~0.997; Table 2) all included an effect of treatment on *S*. Estimated monthly *S* in the dust plot ranged from 0.74 (0.03) to 0.83 (0.04) (Figure 4; Table S2). In the vaccine plot, estimated monthly *S* remained ≥0.81 (0.05) throughout the study (Figure 4; Table S2). In contrast, estimated monthly *S* of prairie dogs in the SNA placebo plot was 0.79 (0.04) between August 2013 and August 2014, but 0.0 (0.0) thereafter (Figure 4; Table S2). As at MSR, *p* and *c* were equal but varied across primary periods, with young having higher capture probabilities than adults (Table S2).

### Flea Abundance

*Yersinia pestis* DNA-positive fleas were most frequent when flea abundance in burrows and on prairie dogs tended to be highest (Figure 2). When flea data from all blocks and years were pooled by treatment, dust plots had fewer fleas per burrow ($\bar{x}$ = 0.1; *P* < 0.0001; Figure 2) than vaccine ($\bar{x}$ = 2.5) or placebo plots ($\bar{x}$ = 2.5). Similarly, prairie dogs captured on dust plots had fewer fleas ($\bar{x}$ = 0.4; *P* < 0.0001) than on vaccine ($\bar{x}$ = 15.8) or placebo plots ($\bar{x}$ = 14). Burrows and prairie dogs on dusted plots had fewer fleas (*P* < 0.05) than vaccine or placebo plots on all blocks and during all years (2013–2015). Differences in flea abundance between vaccine and placebo plots were more variable. At BGA, there was no difference in fleas per burrow on vaccine ($\bar{x}$ = 1.9) and placebo plots ($\bar{x}$ = 0.73; *P* = 0.0860), while fleas were more abundant on prairie dogs on vaccine ($\bar{x}$ = 12.7) than on placebo plots ($\bar{x}$ = 6.3; *P* = 0.0317). At MSR, there were fewer fleas per burrow on vaccine ($\bar{x}$ = 3.7) than on placebo plots ($\bar{x}$ = 5.0; *P* = 0.036) but fleas on prairie dogs were more abundant on vaccine plots ($\bar{x}$ = 19.0) when compared to placebo ($\bar{x}$ = 14.7; *P* = 0.0317). At SNA, there was no difference in fleas per burrow on vaccine ($\bar{x}$ = 2.0) and placebo plots ($\bar{x}$ = 1.8; *P* = 0.980), while fleas were less abundant on prairie dogs on vaccine ($\bar{x}$ = 10.7) than on placebo plots ($\bar{x}$ = 16.3; *P* = 0.0442). See Supplemental Materials for additional flea data.

## Discussion

Vaccination or insecticidal dusting blunted the depressive effects of epizootic plague on prairie dog apparent survival and abundance when compared to data from untreated plots (Figures 2, 3, 4). Although both treatments showed beneficial effects, neither provided complete protection from plague transmission and mortality. Spatial and temporal variation in plague activity across study plots, temporal relationships between plague emergence and respective treatments, our relatively small plot sizes and <100% treatment efficacy likely contributed to the incomplete protection observed.

Plague was present on or adjacent to all nine study plots at one or more time points during our experiment (Figures 1, 2). Moreover, all three placebo plots and about 20 other untreated prairie dog colonies within the Soapstone–Meadow Springs complex collapsed during the course of our study (Figures 1, 2). The duration of plague activity at our study plots (range = 0–26 mo.) is consistent with recent reports of extended plague persistence in prairie dog colonies in Colorado (St. Romain et al. 2013; Salkeld et al. 2016). We detected plague on the BGA vaccine plot on one occasion and on the placebo plot on multiple occasions over 8 months. At MSR, plague was detected on the vaccine plot consistently over 12 months (sporadically for 26 months) and on the placebo plot for 4 months. We detected plague at the SNA vaccine plot on three occasions over 4 months and on multiple occasions over 12 months on the placebo plot. The only plague detection on a dusted plot was a single occasion at SNA (Table 1; Figure 2).

Small treatment plot sizes relative to the overall complex footprint (Figure 1) allowed plague to be transmitted unabated on adjacent colonies and between study plots. This may have compromised apparent vaccine and dust effects. The US Fish and Wildlife Service considers >600 ha of black-tailed prairie dog or >1200 ha of Gunnison’s or white-tailed prairie dog habitat necessary for black-footed ferret reintroduction (USFWS 2013). Moreover, prairie dog complexes >4000 ha appear to be the most productive habitat for reintroduced black-footed ferrets (Jachowski et al. 2011). Our observations underscore the limitations and potential futility of practicing small-scale plague management in the context of black-footed ferret conservation (Tripp et al. 2016). However, our results also demonstrate that plague mitigation on smaller areas may be effective when black-footed ferret recovery is not the primary goal (e.g., prairie dog or associated species conservation).

The relative lack of detected plague activity on the BGA and MSR dust plots (Figure 2) illustrates the potential effectiveness of annual preemptive flea suppression (Figure 1). Comparatively high flea abundance occurred on non-dusted plots in all three blocks throughout our study (Figure 2). Declining prairie dog activity and density on the SNA dust plot between 2014 and 2015 preceded plague detection. Perhaps effects of deltamethrin applied a year earlier had waned (e.g., Tripp et al. 2016). However, plague transmission and mortality on this dust plot occurred despite low detectable flea loads in burrows and on prairie dogs (Figure 2), suggesting the possibility of an alternative form of transmission (e.g., cannibalism, Rust et al. 1972; pneumonic, Gage and Kosoy 2005; louse, Houhamdi et al. 2006; soil, Boegler et al. 2012; multiple, Richgels et al. 2016) or, less likely, a brief but undetected spike in flea abundance. Concurrent mortality from other diseases (e.g., tularemia; La Regina 1986; Avashia et al. 2004) cannot be completely excluded.

The serendipitous timing of plague emergence relative to the start and duration of vaccine delivery across respective plots revealed potential limitations and strengths of vaccination as a management strategy. Plague first impacted the BGA study block in 2013 as baiting began. The BGA vaccine plot’s ensuing collapse was predictable given experimental data showing incomplete protection from plague challenge in prairie dogs vaccinated only 30 days earlier (Rocke et al. 2014). Thus using oral vaccine alone for the first time in the face of epizootic plague should not be expected to suppress plague activity or prevent widespread prairie dog mortality.

Plague also emerged on the MSR vaccine plot (an entire colony, Figure 1) in autumn 2013 (Table 1; Figure 2), but 8 ha (15%) of that colony was vaccinated in autumn 2012 (Tripp et al. 2015). The MSR vaccine colony had fragmented by spring 2014, with only small patches of surviving prairie dogs scattered throughout the original colony footprint. We speculate that most survivors seemed likely to be individuals first vaccinated during the 2012 small plot study (Tripp et al. 2015) and potentially again in 2013. Marked survivors had dispersed (≤1.3 km) away from the location of their original capture in 2012, suggesting that as the colony and its underlying social structure collapsed, the survivors dispersed from their home coteries. Our observation of survivor dispersal in response to collapse of the coterie structure resembled that reported by Hoogland (2013). Dispersal or emigration of survivors may also partially account for the relatively low apparent survival observed at many of our study plots. Although protection was incomplete, recovery of the fragmented MSR vaccine colony was already underway in 2016, while the placebo plot remained unoccupied since collapsing (CPW unpublished data).

Plague also impacted the SNA vaccine plot in 2015, although the two annual vaccinations on this 40.5 ha plot preceding plague emergence appeared relatively effective in protecting prairie dogs. Activity, density and survival of adult prairie dogs on the vaccine plot remained stable during this time, but unvaccinated juveniles born in 2015 did not survive (Figure 3). Given the extent of plague activity surrounding this vaccine plot (Figure 1), observed effects may underestimate efficacy of oral vaccination applied at larger spatial scales. It follows that repeated vaccination of larger areas could provide broader protection to prairie dog colony complexes.

## Conclusions and Management Implications

Burrow dusting and oral vaccination can reduce the impact of epizootic plague in prairie dog colony complexes. Burrow dusting offers more immediate protection by killing fleas and breaking at least some transmission pathways. However, deltamethrin’s effects wane over time and thus annual (especially spring) application does not uniformly guarantee year-round plague suppression. Oral vaccination also can protect prairie dogs from plague and suppress epizootics provided application occurs well in advance of plague emergence. The benefits of annual vaccination, although less immediate than those of burrow dusting, accrue over time.

Maximizing the comprehensive success of plague mitigation in prairie dog colony complexes likely will entail strategic combined uses of burrow dusting and oral vaccination, at least in the near term. Regardless of the specific strategies adopted for using these two tools individually or in combination, treating small plots within larger colonies or small colonies within larger complexes appears unlikely to be effective in suppressing plague. As untreated colonies succumb to plague, infected fleas concentrate on remaining animals, which may overwhelm protection afforded by either vaccine or dust. Similar to the potential advantages of autumn burrow dusting (Tripp et al. 2016), applying vaccine baits in late summer and autumn, when uptake is likely to be higher (Tripp et al. 2014) and juvenile prairie dogs are more likely to become vaccinated (Tripp et al. 2014, 2015; Rocke et al. 2015), may also increase effectiveness of oral vaccination as a plague management tool.

Annual management to mitigate plague and stabilize selected prairie dog populations for conservation purposes will be needed for the foreseeable future. Consequently, we encourage modifying plague management approaches to experimentally incorporate oral vaccination, streamline monitoring and compare preventive treatments in order to develop a sustainable adaptive framework for plague management in selected prairie dog habitats and black-footed ferret recovery areas. Objectives should include developing more practical and versatile methods for vaccine bait production and delivery (e.g., Corro et al. 2017) and assessing the long-term efficacy of oral plague vaccination as part of an integrated plague management strategy for prairie dog conservation in selected locations. How well such efforts translate into stability and growth of prairie dog colonies and persistence of dependent black-footed ferrets are questions of ultimate interest.

## Electronic supplementary material

Below is the link to the electronic supplementary material.

Supplementary material 1 (DOCX 46 kb)
