# Supplementary material for: Burrow Dusting or Oral Vaccination Prevents Plague-Associated Prairie Dog Colony Collapse
Source: Ecohealth. 2017 Jun 22;14(3):451–62. doi: 10.1007/s10393-017-1236-y (PMC5662691; doi:10.1007/s10393-017-1236-y)
Supplement: Supplementary file 1 — Supplementary material 1 (DOCX 46 kb) [file 10393_2017_1236_MOESM1_ESM.docx]

**Annual Burrow Dusting or Oral Vaccination Prevents Plague-Associated Black-Tailed Prairie Dog Colony Collapse**

**Supplemental Material**

**Supplemental Methods**

**Animal Capture & Handling**

Prairie dog capture and handling followed methods described elsewhere (Tripp et al. 2009, 2014, 2015). We captured prairie dogs on at least four consecutive days. The onset of trapping was triggered by bait removal rates and aboveground activity. We used 50−150 Tomahawk traps (Tomahawk Live Trap Co., Tomahawk, Wisconsin, USA) placed individually at randomly selected active burrows and not moved within an annual capture session. Prairie dogs were acclimated by leaving traps wired open and pre-baited for 1−2 weeks with 2−4 applications of 8% 3-way sweet feed (Manna Pro Corp, St. Louis, Missouri, USA). During active trapping we baited and set traps between 0500 and 0700 and checked for captures every 1−2 hours.

We anesthetized captured prairie dogs using isoflurane (Halocarbon Industries, River Edge, New Jersey, USA) via precision vaporizers (Seven-Seven Anesthesia Inc., Fort Collins, Colorado, USA). Once anesthetized, prairie dogs were placed into a clear plastic tub and brushed with a fine-tooth comb to remove fleas. Additionally, whiskers and hair with follicles were plucked to determine bait uptake by prairie dogs (Fernandez and Rocke 2011; Tripp et al. 2014, 2015). Prairie dogs were weighed, classified by sex, age (based on reproductive status, mass, pelage, behavior, and visual comparison of these characteristics to previously marked individuals of known age) and ear-tagged (Monel tag 1005-1; National Band and Tag Co., Newport, Kentucky, USA) and tagged with a passive radio frequency identifier (GPT-12; Biomark Inc., Boise, Idaho, USA). After full recovery from anesthesia, we released each individual at the site of its capture.

**Burrow Swabbing**

Prairie dog burrows were swabbed using methods modified from Ecke and Johnson (1952) as described by Griffin et al. (2010). Briefly, a 1.0cm diameter and 3m length plumbers snake was fitted with an alligator clip to which a 10 cm x 10 cm square white flannel cloth was attached. The cloth was inserted into the burrow until the maximum depth reached. Each burrow was swabbed with two swabs to increase flea detection. Burrow activity (active or inactive) was recorded and the condition of swabbed burrows was used to indicate changes in the prairie dogs’ activity levels (Biggins et al. 1993). All swabs were transferred into sealed plastic bags and returned to the laboratory where fleas were removed after they were frozen.

**Data Analyses**

We used the Huggins robust design model (Huggins 1991) to reduce the number of parameters estimated. For one study area (MSR), sufficient data for young existed to use a multistate robust design (Coffmann and Nichols 1998). Young were assumed to advance to adulthood after one year, thus that probability of transition was set to 1.0. For those models without a multistate component, the probability of emigrating from the plot was set to 0 and the probability of re-entering once an animal left was set to 1. For the robust design, we fit models with 7 different variables based on sex, age (young vs adult in multistate robust design models, first marked as young vs first marked as adult in robust design only models), primary period, treatment (vaccinated, dusted, or control), proportion of colony trapped, proportion of colony treated and colony plague status. However, this model would not converge for any of the blocks due to insufficient data. Consequently, we fit all triple interaction terms and all nested models within them. We also attempted to fit as individual covariates: proportion of colony trapped, proportion of colony that was treated and whether the plot had experienced epizootic plague. Models with these individual covariates either failed to converge or produced identical results to models without the individual covariate and thus were not considered further. Young were assumed to advance to adulthood after one year, thus that probability of transition was set to 1.0 in multistate models. For those models without a multistate component, the probability of temporarily emigrating from the plot was set to 0.

For capture probabilities, the global model would have been an interaction among sex, age, primary period and a behavioral response in which recapture probability differed from capture probability. Data were insufficient to fit time-dependent or heterogeneity-based capture models (Otis et al. 1978). We also used proportion of plot trapped as an individual covariate, but models with this covariate failed to converge.

Fitting all possible models and combinations for both survival and recapture would have resulted in a model set of over 1,000 models, so we used the following steps for model selection:

1. We first reduced the number of terms in the capture probability while keeping the survival model general.
2. We retained the low-AICc model (Akaike 1973, Hurvich and Tsai 1991) for capture probability then reduced the number of terms in the survival model using AICc.
3. Once a low-AICc model was obtained for survival, we also fit competing models for capture probability (all of those models within 10 AICc units of the lowest AICc model after the first step of the process) in combination with all of those survival models within 10 AICc units of the lowest model after step 2.

**Supplemental Results**

**Flea Data by Site**

In all, we collected 12,468 fleas from prairie dog burrows (85% *Oropsylla hirsuta*, 14% *Oropsylla tuberculata cynomuris*, 0.3% *Pulex simulans*, and 0.3% other). We collected 16,121 fleas from captured prairie dogs (73% *O. hirsuta*, 27% *P. simulans*, 0.1% *O. t. cynomuris*, and 0.04% other).

At the BGA study sites, the dusted plot had fewer fleas per burrow (=0.002) than vaccine and placebo plots (=1.3; *P*<0.0001) and fleas per burrow on vaccine plots (=1.9) were not different from placebo plots (=0.73; *P*=0.0860). Dusted plots had fewer fleas per prairie dog (=0.06) than vaccine and placebo plots (=8.0; *P*<0.0001) and there were more fleas per prairie dog on vaccine plots (=12.7) than on placebo plots (=6.3; *P*=0.0317).

At the MSR study sites, the dusted plot had fewer fleas per burrow (=0.002) than vaccine and placebo plots (=4.3; *P*<0.0001) and there were fewer fleas per burrow on vaccine plots (=3.7) than on placebo plots (=5.0; *P*=0.036). Dusted plots had fewer fleas per prairie dog (=0.27) than vaccine and placebo plots (=17.5; *P*<0.0001) and fleas per prairie dog on vaccine plots (=19.0) was greater than from placebo plots (=14.7; *P*=0.0317).

At the SNA study sites, the dusted plot had fewer fleas per burrow (=0.21) than vaccine and placebo plots (=1.9; *P*<0.0001) and fleas per burrow on vaccine plots (=2.0) were not different from placebo plots (=1.8; *P*=0.980). Dusted plots had fewer fleas per prairie dog (=0.8) than vaccine and placebo plots (=12.8; *P*<0.0001) and fleas per prairie dog on vaccine plots (=10.7) were less than from placebo plots (=16.3; *P*=0.0442).

**Flea Data by Site & Year**

In 2013, there were 0.002 fleas per burrow on the dusted plots and 2.0 on vaccine and placebo plots (*P*<0.0001). In 2014, there were 0.2 fleas per burrow on the dusted plots and 4.2 on vaccine and placebo plots *P*<0.0001). In 2015, there were 0.122 fleas per burrow on the dusted plots and 0.895 on vaccine and placebo plots (*P*=<0.0001).

In 2013, there were 0.3 fleas per prairie dog on the dusted plots and 19.9 on vaccine and placebo plots (*P*<0.0001). In 2014, there were 0.6 fleas per prairie dog on the dusted plots and 19.5 on vaccine and placebo plots (*P*<0.0001). In 2015, there were 0.1 fleas per prairie dog on the dusted plots and 5.5 on vaccine and placebo plots (*P*<0.0001).

**Plague-positive Fleas**

At the BGA study sites, plague positive fleas were abundant when flea abundance in burrows and on prairie dogs was highest. On the dust plot, a single plague negative flea was collected from a prairie dog burrow in October 2014. On the vaccine plot fleas per burrow ranged from a low of 0.03 fleas per burrow in October 2014 to a high of 10.8 in August 2013 when 43% of flea pools from burrows and 20% from prairie dogs tested positive for plague. On the placebo plot fleas per burrow ranged from a low of 0.04 in June 2015 to a high of 3.4 fleas per burrow in May of 2014. In March 2014, 30% of flea pools tested positive for plague.

At the MSR study sites, plague positive fleas were abundant when flea abundance in burrows and on prairie dogs was highest. On the dust plot, a single plague negative flea was collected from a prairie dog burrow in August 2013. On the vaccine plot fleas per burrow ranged from a low of 0.15 fleas per burrow in June 2015 to a high of 17.3 in April 2014. In June 2014, 52% of flea pools from burrows and 20% from prairie dogs tested positive for plague. On the placebo plot fleas per burrow ranged from a low of 0.05 in June 2015 to a high of 33.5 fleas per burrow in July of 2014 when 6% of flea pools from burrows tested positive for plague. In March 2014, 30% of flea pools tested positive for plague. In September 2014 four prairie dogs with mean a flea abundance of 62.75 were captured adjacent to the study plot. 6% of these flea pools tested positive for plague.

At the SNA study sites, plague positive fleas were abundant when flea abundance in burrows and on prairie dogs was highest. On the dust plot, fleas per burrow ranged from a low of 0.0 fleas per burrow in May 2015 to a high of 0.74 in March 2015 when 28% of flea pools from burrows tested positive for plague. The largest flea abundance on prairie dogs (1.43) was collected in September 2014. No plague positive fleas were recovered from prairie dogs. On the vaccine plot fleas per burrow ranged from a low of 0.26 fleas per burrow in September 2015 to a high of 5.62 in March 2015 when 5% of the flea pools from burrows tested positive for plague. Plague positive pools were also collected from burrows in March 2014 (21%) and March (5%), May (11%), and July (6%) 2015. The largest flea abundance on prairie dogs (15.7) was collected in September 2014 and plague positive flea pools (11%) were collected from prairie dogs in June 2015. On the placebo plot fleas per burrow ranged from a low of 0.33 in May 2013 to a high of 7.8 fleas per burrow in July of 2014 when 4% of flea pools from burrows tested positive for plague. Plague positive pools were also collected from burrows in March (11%) and July (4%) 2014 and March (11%), May (44%), and July (26%) 2015. The largest flea abundance on prairie dogs (18.4) was collected in September 2014 and plague positive flea pools (75%) were collected from prairie dogs in June 2015.

**Mark Recovery and Trap Success**

Mark recovery is defined as the % of marked prairie dogs that were recaptured during the following primary capture session. Trap success is defined as the total captures/total trap days.

Mark recovery and trap success were compared by treatment within each block (Table S3). We compared mark recovery and trap success between treatments using *χ*^2^ tests. The R statistical package (prop.test) was used for these analyses (R Development Core Team, Version 2.15.2, 2012).

**Supplemental Table 1.** Prairie Dog Density Per Hectare Estimates and 95% Confidence Intervals for (A) Bulger Grazing Allotment (BGA), (B) Meadow Springs Ranch (MSR), and (C) and Soapstone Natural Area (SNA) Study Blocks. ns= not sampled. nc= not calculated.

| **A. Treatment** | **Interval/Age** | **Density (Ha)** | **SE** | **95% CI** |
| --- | --- | --- | --- | --- |
| **BGA** |  |  |  |  |
| Dust | 2013 Adult | 3.93 | 3.14 | 0.99-15.59 |
| Dust | 2013 Adult | 4.02 | 2.27 | 1.44-11.25 |
| Dust | 2014 Adult | 1.43 | 0.23 | 1.05-1.95 |
| Dust | 2015 Adult | 8.16 | 2.1 | 4.96-13.4 |
| Dust | 2013 Young | 1.31 | 1.11 | 0.31-5.55 |
| Dust | 2013 Young | 0.57 | 0.55 | 0.12-2.77 |
| Dust | 2014 Young | 4.68 | 0.54 | 3.73-5.87 |
| Dust | 2015 Young | 5.93 | 1.69 | 3.43-10.26 |
| Vaccine | 2013 Adult | 1.78 | 1.52 | 0.42-7.59 |
| Vaccine | 2013 Adult | 0 | 0 | nc |
| Vaccine | 2014 Adult | 0.23 | 0.07 | 0.13-0.42 |
| Vaccine | 2015 Adult | 0.53 | 0.51 | 0.11-2.56 |
| Vaccine | 2013 Young | 0.67 | 0.8 | 0.11-4.25 |
| Vaccine | 2013 Young | 0 | 0 | nc |
| Vaccine | 2014 Young | 0 | 0 | nc |
| Vaccine | 2015 Young | 3.2 | 1.33 | 1.47-6.98 |
| Placebo | 2013 Adult | 5.12 | 4.09 | 1.29-20.31 |
| Placebo | 2013 Adult | 2.05 | 1.32 | 0.65-6.5 |
| Placebo | 2014 Adult | 0.17 | 0.06 | 0.08-0.34 |
| Placebo | 2015 Adult | 5.75 | 2.03 | 2.93-11.26 |
| Placebo | 2013 Young | 2.23 | 1.95 | 0.51-9.76 |
| Placebo | 2013 Young | 2.05 | 1.32 | 0.65-6.5 |
| Placebo | 2014 Young | 0.33 | 0.09 | 0.19-0.57 |
| Placebo | 2015 Young | 1.28 | 0.88 | 0.38-4.35 |
| **B. Treatment** | **Interval/Age** | **Density (Ha)** | **SE** | **95% CI** |
| **MSR** |  |  |  |  |
| Dust | 2012 Adult | ns | ns | ns |
| Dust | 2013 Adult | 3.19 | 0.15 | 2.91-3.49 |
| Dust | 2014 Adult | 1.92 | 0.2 | 1.57-2.34 |
| Dust | 2015 Adult | 1.17 | 0.11 | 0.98-1.41 |
| Dust | 2012 Young | ns | ns | ns |
| Dust | 2013 Young | 1.15 | 0.09 | 0.98-1.35 |
| Dust | 2014 Young | 2.98 | 0.35 | 2.37-3.75 |
| Dust | 2015 Young | 1.04 | 0.25 | 0.66-1.64 |
| Vaccine | 2012 Adult | 9.94 | 1.5 | 7.41-13.34 |
| Vaccine | 2013 Adult | 4.49 | 0.31 | 3.91-5.14 |
| Vaccine | 2014 Adult | 0.82 | 0.03 | 0.75-0.89 |
| Vaccine | 2015 Adult | 0.79 | 0.07 | 0.67-0.93 |
| Vaccine | 2012 Young | 7.46 | 1.84 | 4.63-12.02 |
| Vaccine | 2013 Young | 4.14 | 0.34 | 3.52-4.86 |
| Vaccine | 2014 Young | 0.3 | 0.03 | 0.24-0.36 |
| Vaccine | 2015 Young | 0.19 | 0.07 | 0.1-0.38 |
| Placebo | 2012 Adult | 15.51 | 2.37 | 11.52-20.88 |
| Placebo | 2013 Adult | 4.98 | 0.33 | 4.37-5.66 |
| Placebo | 2014 Adult | 0 | 0 | nc |
| Placebo | 2015 Adult | 0 | 0 | nc |
| Placebo | 2012 Young | 0.93 | 0.53 | 0.33-2.61 |
| Placebo | 2013 Young | 2.42 | 0.24 | 1.98-2.94 |
| Placebo | 2014 Young | 0 | 0 | nc |
| Placebo | 2015 Young | 0 | 0 | nc |
| **C. Treatment** | **Interval/Age** | **Density (Ha)** | **SE** | **95% CI** |
| **SSN** |  |  |  |  |
| Dust | 2013 Adult | 1.58 | 0.16 | 1.29-1.93 |
| Dust | 2014 Adult | 1.28 | 0.12 | 1.07-1.54 |
| Dust | 2015 Adult | 0.76 | 0.1 | 0.59-0.99 |
| Dust | 2015 Adult | 0.64 | 0.03 | 0.58-0.7 |
| Dust | 2013 Young | 5.26 | 0.74 | 4-6.92 |
| Dust | 2014 Young | 6.18 | 0.22 | 5.76-6.64 |
| Dust | 2015 Young | 1.57 | 0.15 | 1.3-1.89 |
| Dust | 2015 Young | 1.55 | 0.02 | 1.51-1.58 |
| Vaccine | 2013 Adult | 1.27 | 0.13 | 1.04-1.56 |
| Vaccine | 2014 Adult | 0.94 | 0.1 | 0.76-1.16 |
| Vaccine | 2015 Adult | 1.36 | 0.14 | 1.11-1.65 |
| Vaccine | 2015 Adult | 1.65 | 0.05 | 1.56-1.76 |
| Vaccine | 2013 Young | 2.63 | 0.43 | 1.91-3.63 |
| Vaccine | 2014 Young | 3.69 | 0.18 | 3.36-4.05 |
| Vaccine | 2015 Young | 0.5 | 0.07 | 0.38-0.67 |
| Vaccine | 2015 Young | 0.55 | 0.01 | 0.53-0.57 |
| Placebo | 2013 Adult | 2.08 | 0.19 | 1.74-2.48 |
| Placebo | 2014 Adult | 0.67 | 0.08 | 0.54-0.84 |
| Placebo | 2015 Adult | 0.05 | 0.02 | 0.02-0.11 |
| Placebo | 2015 Adult | 0 | 0 | nc |
| Placebo | 2013 Young | 0.53 | 0.17 | 0.28-0.98 |
| Placebo | 2014 Young | 3.29 | 0.15 | 3.01-3.61 |
| Placebo | 2015 Young | 0.05 | 0.02 | 0.02-0.11 |
| Placebo | 2015 Young | 0 | 0 | nc |

**Supplemental Table 2.** Survival (A) and Capture/Recapture Probability (B) Results from the Bulger Grazing Allotment (BGA), Meadow Springs Ranch (MSR), and Soapstone Natural Area (SNA) Study Blocks. ns= not sampled. nc= not calculated.

| **A. Treatment** | **Interval** | **Monthly Survival** | **SE** | | **95% CI** | |  |
| --- | --- | --- | --- | --- | --- | --- | --- |
| **BGA** |  |  |  | |  | |  |
| Pooled | 2013-2013 | 0.55 | 0.24 | | 0.16-0.89 | |  |
|  | 2013-2014 | 0.82 | 0.04 | | 0.72-0.89 | |  |
|  | 2014-2015 | 1 | 0 | | 1.00-1.00 | |  |
| **MSR** |  |  |  | |  | |  |
| Dust | 2012-2013 | 0 | 0 | | ns | |  |
|  | 2013-2014 | 0.87 | 0.02 | | 0.83-0.90 | |  |
|  | 2014-2015 | 0.81 | 0.03 | | 0.76-0.86 | |  |
| Vaccine | 2012-2013 | 0.89 | 0.02 | | 0.84-0.92 | |  |
|  | 2013-2014 | 0.79 | 0.02 | | 0.75-0.83 | |  |
|  | 2014-2015 | 0.92 | 0.02 | | 0.88-0.95 | |  |
| Placebo | 2012-2013 | 0.91 | 0.02 | | 0.86-0.94 | |  |
|  | 2013-2014 | 0 | 0 | | nc | |  |
|  | 2014-2015 | 0 | 0 | | nc | |  |
| **SNA** |  |  |  | |  | |  |
| Dust | 2013-2014 | 0.81 | 0.03 | | 0.76-0.86 | |  |
|  | 2014-2015 | 0.74 | 0.03 | | 0.69-0.79 | |  |
|  | 2015-2015 | 0.83 | 0.04 | | 0.72-0.90 | |  |
| Vaccine | 2013-2014 | 0.83 | 0.03 | | 0.76-0.88 | |  |
|  | 2014-2015 | 0.82 | 0.02 | | 0.77-0.86 | |  |
|  | 2015-2015 | 0.81 | 0.05 | | 0.70-0.89 | |  |
| Placebo | 2013-2014 | 0.79 | 0.04 | | 0.69-0.86 | |  |
|  | 2014-2015 | 0 | 0 | | nc | |  |
|  | 2015-2015 | 0 | 0 | | nc | |  |
|  |  |  |  | |  | |  |
|  |  |  |  | |  | |  |
| **B. Parameter/Age** | **Primary Period** | **Estimate** | | **SE** | | **95% CI** | |
| **BGA** |  |  | |  | |  | |
| Capture/Pooled | 2013 | 0.06 | | 0.1 | | 0.00-0.49 | |
|  | 2013 | 0.11 | | 0.1 | | 0.02-0.41 | |
|  | 2014 | 0.28 | | 0.1 | | 0.17-0.43 | |
|  | 2015 | 0.03 | | 0 | | 0.02-0.05 | |
| Recapture/Pooled | 2013 | 0.16 | | 0 | | 0.11-0.23 | |
|  | 2013 | 0 | | 0 | | 0.00-0.00 | |
|  | 2014 | 0.26 | | 0 | | 0.20-0.33 | |
|  | 2015 | 0.37 | | 0.1 | | 0.28-0.47 | |
| **MSR** |  |  | |  | |  | |
| Capture&Recapt./Adult | 2012 | 0.07 | | 0 | | 0.05-0.10 | |
|  | 2013 | 0.25 | | 0 | | 0.22-0.28 | |
|  | 2014 | 0.24 | | 0 | | 0.21-0.28 | |
|  | 2015 | 0.29 | | 0 | | 0.23-0.36 | |
| Capture&Recapt./Young | 2012 | 0.06 | | 0 | | 0.03-0.12 | |
|  | 2013 | 0.24 | | 0 | | 0.19-0.28 | |
|  | 2014 | 0.19 | | 0 | | 0.14-0.25 | |
|  | 2015 | 0.15 | | 0.1 | | 0.08-0.27 | |
| **SNA** |  |  | |  | |  | |
| Capture&Recapt./Adult | 2013 | 0.24 | | 0 | | 0.20-0.29 | |
|  | 2014 | 0.11 | | 0 | | 0.08-0.16 | |
|  | 2015 | 0.35 | | 0 | | 0.28-0.43 | |
|  | 2015 | 0.38 | | 0 | | 0.34-0.41 | |
| Capture&Recapt./Young | 2013 | 0.28 | | 0 | | 0.21-0.37 | |
|  | 2014 | 0.29 | | 0 | | 0.22-0.37 | |
|  | 2015 | 0.5 | | 0 | | 0.43-0.57 | |
|  | 2015 | 0.67 | | 0 | | 0.60-0.73 | |
|  |  |  | |  | |  | |

**Supplemental Table 3.** Mark Recovery (A) and Trap Success (B) Results from the Bulger Grazing Allotment (BGA), Meadow Springs Ranch (MSR), and Soapstone Natural Area (SNA) Study Blocks. We compared treatments using *χ*^2^ tests.

| **A. Mark Recovery** | **Treatment Comparison** | **Treatment 1(%)** | **Treatment 2(%)** | ***P*-value** |
| --- | --- | --- | --- | --- |
| **BGA** | **Treatment 1/Treatment 2** | |  |  |
| 2014 | Vaccine/Placebo | 0.00 | 0.00 | na |
|  | Dust/ Vaccine | 13.16 | 0.00 | 0.4814 |
|  | Dust /Placebo | 13.16 | 0.00 | 0.0463 |
| 2015 | Vaccine/Placebo | 7.14 | 6.12 | 1.0000 |
|  | Dust/ Vaccine | 9.65 | 7.14 | 1.0000 |
|  | Dust /Placebo | 9.65 | 6.12 | 0.6658 |
| **MSR** |  |  |  |  |
| 2014 | Vaccine/Placebo | 5.63 | 0.00 | 0.0054 |
|  | Dust/ Vaccine | 15.46 | 5.63 | 0.0041 |
|  | Dust /Placebo | 15.46 | 0.00 | <0.0001 |
| 2015 | Vaccine/Placebo | 6.67 | 0.00 | 0.0093 |
|  | Dust/ Vaccine | 5.33 | 6.67 | 0.7095 |
|  | Dust /Placebo | 5.33 | 0.00 | 0.0284 |
| **SNA** |  |  |  |  |
| 2014 | Vaccine/Placebo | 8.33 | 4.36 | 0.6713 |
|  | Dust/ Vaccine | 6.25 | 8.33 | 0.8870 |
|  | Dust /Placebo | 6.25 | 4.36 | 0.9642 |
| 2015 | Vaccine/Placebo | 8.90 | 0.00 | 0.0011 |
|  | Dust/ Vaccine | 1.43 | 8.90 | 0.0020 |
|  | Dust /Placebo | 1.43 | 0.00 | 0.4041 |
| **B. Trap Success** | **Treatment Comparison** | **Treatment 1(%)** | **Treatment 2(%)** | **p-value** |
| **BGA** | **Treatment 1/Treatment 2** | |  |  |
| 2013 | Vaccine/Placebo | 1.57 | 6.14 | <0.0001 |
|  | Dust/ Vaccine | 5.46 | 1.57 | 0.0001 |
|  | Dust /Placebo | 5.46 | 6.14 | 0.6662 |
| 2014 | Vaccine/Placebo | 1.50 | 3.00 | 0.5001 |
|  | Dust/ Vaccine | 20.25 | 1.50 | <0.0001 |
|  | Dust /Placebo | 20.30 | 3.00 | <0.0001 |
| 2015 | Vaccine/Placebo | 2.80 | 4.40 | 0.4714 |
|  | Dust/ Vaccine | 7.60 | 2.80 | 0.0144 |
|  | Dust /Placebo | 7.60 | 4.40 | 0.1298 |
| **MSR** |  |  |  |  |
| 2013 | Vaccine/Placebo | 24.67 | 21.67 | 0.2448 |
|  | Dust/ Vaccine | 9.24 | 24.70 | <0.0001 |
|  | Dust /Placebo | 9.24 | 21.70 | <0.0001 |
| 2014 | Vaccine/Placebo | 3.58 | 0.00 | 0.0015 |
|  | Dust/ Vaccine | 21.25 | 3.58 | <0.0001 |
|  | Dust /Placebo | 21.30 | 0.00 | <0.0001 |
| 2015 | Vaccine/Placebo | 5.38 | 0.00 | <0.0001 |
|  | Dust/ Vaccine | 8.00 | 5.38 | 0.0969 |
|  | Dust /Placebo | 8.00 | 0.00 | <0.0001 |
| **SNA** |  |  |  |  |
| 2013 | Vaccine/Placebo | 12.00 | 8.80 | 0.1144 |
|  | Dust/ Vaccine | 16.00 | 12.00 | 0.0834 |
|  | Dust /Placebo | 16.00 | 8.80 | 0.0006 |
| 2014 | Vaccine/Placebo | 22.75 | 22.75 | 1.0000 |
|  | Dust/ Vaccine | 33.75 | 22.80 | 0.0007 |
|  | Dust /Placebo | 33.75 | 22.80 | 0.0007 |
| 2015 | Vaccine/Placebo | 9.40 | 0.00 | <0.0001 |
|  | Dust/ Vaccine | 3.60 | 9.40 | 0.0003 |
|  | Dust /Placebo | 3.60 | 0.00 | 0.0001 |
